# Deterministic processes influence bacterial more than fungal community assembly during the development of biological soil crusts in the desert ecosystem

**DOI:** 10.3389/fmicb.2024.1404602

**Published:** 2024-08-23

**Authors:** Hong Zhou, Ke Yu, Chunfang Deng, Bo Wu, Ying Gao

**Affiliations:** ^1^Academy of Agriculture and Forestry Sciences, Qinghai University, Xining, China; ^2^Institute of Ecological Conservation and Restoration, Chinese Academy of Forestry, Beijing, China; ^3^Qinghai Guinan Desert Ecosystem Positioning Observation and Research Station, National Forestry and Grassland Administration, Beijing, China; ^4^Key Laboratory of Desert Ecosystem and Global Change, State Administration of Forestry and Grassland, Beijing, China; ^5^School of Environment and Energy, Shenzhen Graduate School, Peking University, Shenzhen, China; ^6^Institute of Desertification Studies, Chinese Academy of Forestry, Beijing, China

**Keywords:** biological soil crusts, bacterial and fungal communities, community assembly, Illumina sequencing, semi-arid regions

## Abstract

Biological soil crusts (biocrusts) constitute a crucial biological component of the soil surface in arid and semi-arid ecosystems. Understanding the variations in soil microbial community assembly across biocrust successional stages is essential for a deeper comprehension of microbial biodiversity and desert ecosystem functioning. However, knowledge about the mechanisms of microbial community assembly and the factors influencing its development remains limited. In this study, we utilized amplicons sequencing to assess the compositions of bacterial and fungal communities in bare sand and three types of biocrusts (light cyanobacterial biocrusts, dark cyanobacterial biocrusts, and moss crusts). Subsequently, we analyzed the ecological processes shaping microbial community composition and structure, along with the influencing factors. Our results revealed a significant increase in bacterial diversity and no significant changes in fungal diversity during biocrust development. The relative abundances of the copiotrophic bacteria (e.g., Actinobacteria, Acidobacteria, and Bacteroidetes) showed significant increases, while oligotrophic bacteria (e.g., Proteobacteria and Firmicutes) decreased over time. Moreover, the relative abundances of Ascomycota, which exhibit strong resistance to adverse environmental conditions, significantly decreased, whereas Basidiomycota, known for their ability to degrade lignin, significantly increased throughout biocrust development. Additionally, stochastic processes (dispersal limitation and drift) predominantly drove the assemblies of both bacterial and fungal communities. However, the relative importance of deterministic processes (homogeneous selection) in bacterial assembly increased during biocrust development. Structural equation modeling indicated that bacterial community assembly was primarily related to soil water content, whereas fungal community assembly was primarily related to total organic carbon. These findings provide a scientific foundation for investigating the formation and development of biocrusts, and further insights into the conservation and sustainable management of biocrust resources under future climate change scenarios.

## 1 Introduction

Biological soil crusts (biocrusts) cover the top layers of the ground as coherent layer formed by soil particles closely associated with different proportions of photoautotrophic (e.g., cyanobacteria, algae, lichens, bryophytes) and heterotrophic (e.g., bacteria, fungi, archaea) organisms (Lan et al., 2012; Weber et al., 2022). They cover approximately 40% of the soil surfaces in arid and semi-arid regions, which are characterized by limited precipitation, intense evaporation, sparse vegetation, and strong solar radiation (Yeager et al., 2004; Ferrenberg et al., 2015). Indeed, they are recognized as engineers of desert ecosystems, playing crucial roles in stabilizing sand dunes, preventing wind-induced soil erosion, and regulating water and soil nutrient cycling (Belnap and Weber, 2013; Zhou et al., 2020).

The development of biocrusts is a dynamic process that begins with the colonization of the soil surface by cyanobacteria (Belnap and Lange, 2003; Zaady et al., 2010; Lan et al., 2013), followed by successive colonization by diazotrophic and photosynthetic cyanobacteria, fungi, lichens, and mosses over time (Maier et al., 2018). Depending on the dominant taxa (cyanobacteria, lichens, or mosses), biocrusts can be mainly classified into cyanobacterial, lichen, and moss-dominated biocrusts, representing different successional stages of soil micro-environments/ecosystems (Bowker et al., 2014). The succession of biocrusts may alter soil aggregate stability, as well as physical, chemical, and biochemical properties in the underlying layers (Yang et al., 2022). Microbial species abundances and compositions within biocrusts can significantly vary across different successional stages. Previous research has highlighted Cyanobacteria as the predominant phylum in cyanobacterial biocrusts (Liu L. C. et al., 2017; Liu Y. R. et al., 2017; Nuttapon et al., 2020). In early biocrust successional stages, motile non-heterocystous cyanobacteria, such as *Microcoleus vaginatus* or *M. steenstrupii*, are considered as the pioneer colonizers (Weber et al., 2016). These species are capable of forming centimeter-long filament bundles, thereby contributing to the maintenance of the physical structure of biocrusts (Garcia-Pichel and Wojciechowsk, 2009). As the biocrusts transition to mature, the dominant species shift to the non-motile N_2_-fixing heterocystous cyanobacteria, such as *Scytonema*, *Tolypothrix* and *Nostoc*. These genera produce large amounts of sunscreen compounds, which reduce soil albedo, thereby providing the dark color to more mature and forming dark cyanobacterial biocrusts (Yeager et al., 2010; Muñoz-Martín et al., 2019). Once the soil fertility is enriched by cyanobacteria and soil surface stabilizes, mosses begin to emerge. This is particularly evident in arid and semiarid regions, where species like *Bryum argenteum*, *Syntrichia* spp., and *Tortula* spp. thrive (Wu et al., 2014). These species exhibit densely packed stems or leaves, which form a compact growth pattern. This structural feature enhances the boundary layer resistance of the cuticle, optimizing water transfer and absorption while concurrently minimizing water loss (Zhang et al., 2009). Concurrently, in moss crusts, Actinobacteria emerge as a dominant group (Zhou et al., 2020), contributing to the degradation of complex compounds, such as polysaccharides and phenolic compounds, thereby enhancing the nutritional status of the biocrusts. Moreover, Actinobacteria genera like *Actinoplanes*, *Pseudonocardia*, and *Streptomyces*, characterized by their mycelial structures, are vital for maintaining the structural integrity of moss crusts (Maier et al., 2016). Across all stages of biocrust development, Ascomycota is identified as a key fungal phylum (Bates and Garcia-Pichel, 2009; Zhou et al., 2020). However, at finer taxonomic levels, such as class and genus, noticeable changes occur during different developmental stages. For example, Zhang B. C. et al. (2018) noted that the prevalence of *Dothideomycetes*, *Eurotiomycetes*, and *Lecanoromycetes* in cyanobacterial crusts, while *Ascomycetes* and *Eurotiomycetes* were predominant in moss crusts in the Gurbantunggut Desert, China. The *Dothideomycetes* and *Eurotiomycetes* share common characteristics of adaptation or resistance to low temperature and drought, which may be essential for adapting to environment in arid region (Nagano and Nagahama, 2012). While many studies have assessed microbial community composition across biocrust developmental stages (Abed et al., 2013; Zhang B. C. et al., 2018; Zhang Q. Y. et al., 2018), few have examined microbial community assembly in biocrusts.

Microbial community assembly processes are pivotal in governing the construction of microbial communities, and the dynamics of these assembly processes influence species coexistence and the maintenance of functional diversity (Zhou and Ning, 2017). These processes are influenced by both deterministic factors (such as selection mediated by biotic and abiotic factors) and stochastic ecological processes (including random dispersal and drift events), which often act concurrently (Bell, 2010; Green and Bohannan, 2006). A small number of studies have explored the effects of geographical distance and environmental variations on microbial assembly in biocrusts at the landscape scales (Wei et al., 2018; Ding et al., 2023). According to Su et al. (2020), environmental variables exert significant impacts on bacterial communities within biocrusts, with precipitation, water content, and nutrient availability playing crucial roles in community assembly. In contrast, Li and Hu (2021) have recorded that stochastic processes predominantly influence fungal communities, as evidenced by distance-decay relationships. However, there is a notable gap in understanding the substantial influence that distinct biocrust developmental stage exert on the assembly processes of microbial communities at smaller spatial scales. Furthermore, previous studies have only broadly classified community assembly processes without quantitatively analyzing the contributions of stochastic and deterministic processes at different stages of biocrust development. The distinct microbial communities in different developmental stage of biocrusts could significantly affect the response of desert soil functionality to global change (Liu L. C. et al., 2017; Liu Y. R. et al., 2017). Thus, given the importance of biocrusts for the sustainability of global drylands, understanding the variation patterns and assembly processes of microbial communities is crucial for predicting the response of ecosystem functions to ongoing global changes.

In this study, we employed 16S rRNA and internal transcribed spacer (ITS 1) gene amplicon sequencing to explore the diversity, composition, and assembly of microbial communities within biocrusts at different developmental stages in the Mu Us Sandy Land, located in the Inner Mongolian Autonomous Region, northwestern China. Our primary objectives were to investigate differences in microbial composition, assembly processes, and physicochemical properties across different developmental stages of biocrusts. To address these aims, we hypothesize that: (i) deterministic processes will predominantly govern the assembly of bacterial and fungal communities in all developmental stages of biocrusts, and (ii) soil moisture might influence the assembly of bacterial and fungal communities during the development of biocrusts.

## 2 Materials and methods

### 2.1 Study site

The study was conducted in Uxin Banner County, located at a latitude of 38°28′N, a longitude of 108°42′E, and an altitude of 1,306 m, respectively, in the central part of the Mu Us Sandy Land in the Inner Mongolian Autonomous Region, China (Supplementary Figure S1). The Mu Us sandy land lies in the transitional zone between typical steppes to deserts. It has a semi-arid continental climate. The mean annual temperature in the study area is 8.4°C, while the average annual precipitation is 341 mm, with 70% occurring during the July–September period (Wu and Ci, 2002). The study area is characterized by a sandy soil texture (Chen et al., 1998). The main vegetation types in the research area consist of sparse shrub species, including *Artemisia ordosica Krasch*, *Caragana korshinskii, Sabina vulgaris,* and *Salix cheilophila*, covering approximately 30–50%, 10–30, and <10% of the fixed, semi-fixed, and shifting sand dunes, respectively (Zhang, 1994). In addition, different biocrust types commonly cover the open areas between the shrubs in the fixed and semi-fixed dunes, with cyanobacterial, and moss crusts being the most prevalent (Zhang, 1994). The light cyanobacterial biocrusts are generally weakly stabilized by *Microcoleus vaginatus*, with no or low levels of the darker-pigmented cyanobacterial species. In contrast, the relatively dark cyanobacterial biocrusts are dominated by *M. vaginatus* but also contain other smaller, darker-pigmented cyanobacteria, such as *Scytonema myochrous* and *Nostoc* (a genus of nitrogen-fixing cyanobacteria with a relative abundance of 7.9% in this study) (Muñoz-Martín et al., 2019). The moss crusts in the study area are mainly composed of drought-tolerant Bryum species, including *Bryum pallescen*, *Bryum recurvulum*, and a small amount of *Bryum argenteu*.

### 2.2 Sampling and analysis of the biocrusts

Samples were collected on August 2019. Eight 12.5 m × 100 m plots, with eight replicates, were selected in a relatively flat area of fixed sand dunes to explore the microbial community compositions in the selected biocrusts (Supplementary Figure S2). Replicate plots were spaced at a distance of more than 5 m from each other. Samples of the three main biocrusts in this study, namely light cyanobacterial biocrusts, dark cyanobacterial biocrusts, and moss crusts, were randomly collected from the interspaces between shrubs in each selected plot. From each plot, five individual biocrust samples were collected and then mixed to obtain a composite sample. Specifically, pieces of biocrusts (9 cm diameter) in their natural thickness (4–15 mm), without the underlying soil, were carefully sampled along the polygonal cracking sections using a sharp shovel and then mixed in a plastic bag. Bare sand samples were collected from the shifting sand dunes near the fixed sand dunes. A total of 32 samples (4 stages × 8 replicates) were collected from these dunes. The collected samples were preserved in an ice box and transported to the laboratory, where they were immediately sieved through a 2 mm sieve to remove stones, plant roots, and mosses. The soil samples were divided into three parts. The first part was stored at 4°C and analyzed for soil water contents (SWC), nitrate (NO_3_^−^) contents, ammonium (NH_4_^+^) contents, soil microbial biomass C (MBC), and soil microbial biomass N (MBN) within 48 h. The second part was air-dried and analyzed for other soil physicochemical parameters, namely total carbon (TC), total nitrogen (TN), total phosphorous (TP), total organic carbon (TOC), and soil pH. The last part was stored at -80°C and analyzed for soil DNA.

Soil water content (SWC) was measured using the gravimetric method at 105°C. Soil pH was measured in a soil suspension at a soil: water ratio of 1:2.5 (w/v) using a pH electrode (Leici, Shanghai, China). Soil NO_3_^−^ and NH_4_^+^ contents were extracted using a 50 mL solution of 2 M KCl. The solution was shaken for 30 min, then filtered and analyzed using a continuous flow analyzer (Skalar, Breda, Netherlands). MBC and MBN were determined using the chloroform fumigation direct extraction method (Vance et al., 1987), while TC and TOC contents were determined using a total carbon analyzer (vario TOC, Elementar, Germany). TN contents were determined using the semi-micro Kjeldahl method (UDK140 Automatic Steam Distilling Unit, Automatic Titroline 96, Italy), while TP contents were determined using a UV-2550 spectrophotometer (Shimadzu, Kyoto, Japan) after H_2_SO_4_–HClO_4_ digestion.

### 2.3 DNA extraction, PCR amplification, and sequencing

In this study, DNA was extracted from 0.5 g of biocrust using the MoBio PowerSoil^®^ DNA isolation kit (MO BIO Laboratories, Carlsbad, CA, United States), following the manufacturer’s instructions. The quality of the extracted DNA was evaluated based on 260/280 nm (>1.8) and 260/230 nm (>1.7) ratios, which were obtained using a NanoDrop ND-1000 spectrophotometer (NanoDrop Technologies Inc., DE, United States). The final DNA concentration was quantified using a Quant-IT Pico Green dsDNA Kit (Invitrogen Molecular Probes Inc., Oregon, United States). A Polymerase Chain Reaction (PCR) analysis was performed on the extracted DNA using a barcoded primer set (515F and 806R) designed to capture the V4 region of the 16S ribosomal RNA genes of the bacterial and archaeal communities. Primers ITS5-1737F and ITS2-2043R were used to amplify the ITS1 region of the fungal rRNA gene. The PCR products for each sample were first combined, then amplicons from different samples were pooled in equimolar concentrations and sequenced using an Illumina MiSeq platform (Illumina Inc., San Diego, CA, United States) at the Novogene Bioinformatics Technology Co., Ltd. (Beijing, China). Paired-end reads in 2 × 300 bp format were generated using the FLASH program (Reyon et al., 2012). A total of 2,685,761 and 2,514,026 paired-end sequence reads of bacteria and fungi were generated and introduced in the NCBI Sequence Read Archive (SRA) databases associated with BioProject accession numbers PRJNA553985 and PRJNA554507, respectively. In addition, QIIME 2 was used to analyze high-quality reads, as detailed in the Supplementary material.

### 2.4 Statistical analysis

In this study, differences in the microbial diversity index, abundance, and soil physicochemical characteristics among the four stages were assessed using the one-way analysis of variance (ANOVA) test. In addition, *post hoc* analyses were performed using Fisher’s least significant difference (LSD) test. Differences at the *p* < 0.05 level were considered statistically significant. Differences in the microbial community composition (Bray–Curtis dissimilarity) among the four stages were assessed using permutational multivariate ANOVA (PERMANOVA) and visualized using Principal Coordinate Analysis (PCoA). The relationships between the microbial community compositions of biocrusts and physicochemical properties of the biocrusts (SWC, NO_3_^−^, NH_4_^+^, TC, TN, TP, TOC, and soil pH) were assessed using Mantel test. The Infer Community Assembly Mechanisms by the Phylogenetic-bin-based null model (iCAMP) was applied to investigate the assembly mechanisms of the microbial groups (Ning et al., 2020) using the *iCAMP* R package downloaded from the Comprehensive R Archive Network (CRAN^1^). The Beta Nearest Taxon Index (Beta NTI) was also calculated using the R “iCAMP” package. All statistical tests were performed using the vegan package (Oksanen et al., 2016) in R 3.6.0, while graphs were generated in this study using the *ggplot2* package (Wickham, 2009).

Structural equation models (SEM), using AMOS graphic (Zhang et al., 2021), comprised physicochemical properties of biocrusts, as direct and indirect drivers of microbial community assembly (as assessed by based on Beta NTI values). In contrast to ANOVA tests, which primarily concentrate on the central tendencies of individual variables, SEM analysis is a multivariate statistical analysis technique that emphasizes the overall relationships and structures among the variables under analysis, revealing significant interactions or indirect effects (Bagozzi and Yi, 2012; Hsu et al., 2015). The combined utilization of these two methods enhances our understanding of the complex relationships between physicochemical properties and microbial communities in biocrusts. Initially, we standardized (Z-score) the individual parameters using SPSS and then entered into AMOS graphic application. Accordingly, we assumed that (1) soil water and conditions (e.g., TC, TN, and TOC) directly affected the bacterial and fungal community assembly, or indirectly through changing affecting each other; (2) SEM of drivers of bacterial and fungal communities would differ during the biocrust development, based on our ANOVA and Mantel test results. We accepted the SEMs at *p* > 0.05, with high levels of goodness of fit index (>0.9), and root mean square error of approximation (<0.05).

## 3 Results

### 3.1 Physicochemical properties of the biocrusts

The results of this study indicated that the contents of SWC, TOC, TC, TN, and TP in the 3 types of biocrusts were significantly higher than those in bare sand, exhibiting a gradual increase with biocrust development (Supplementary Table S1). Specially, from bare sand to moss crusts, the contents of SWC, TOC, TC, and TN contents significantly increased by 4, 23.2, 8.6, and 3 times, respectively. Moreover, soil NH_4_^+^ contents increased by 80%, whereas NO_3_^−^ contents and soil pH values significantly decreased by 68.5 and 2.7%, respectively (*p* < 0.05). In addition, the measured silt and clay contents increased from 4.1 to 17.1% and 0.5 to 1.9%, respectively, with the biocrust development. In contrast, the observed sand content decreased from 95.4 to 81% with the biocrust development. The measured MBC and MBN in biocrusts were significantly higher than those in bare sand and showed a significant increase with biocrust development (Supplementary Figure S3). Specifically, from light cyanobacterial to moss crusts, MBC and MBN values increased from 137.46 ± 31.78 to 389.80 ± 26.16 mg·kg^−1^ and 13.96 ± 2.24 to 39.42 ± 4.36 mg·kg^−1^, respectively.

### 3.2 Microbial community diversity and compositions in different biocrust types

**Figure 1:**
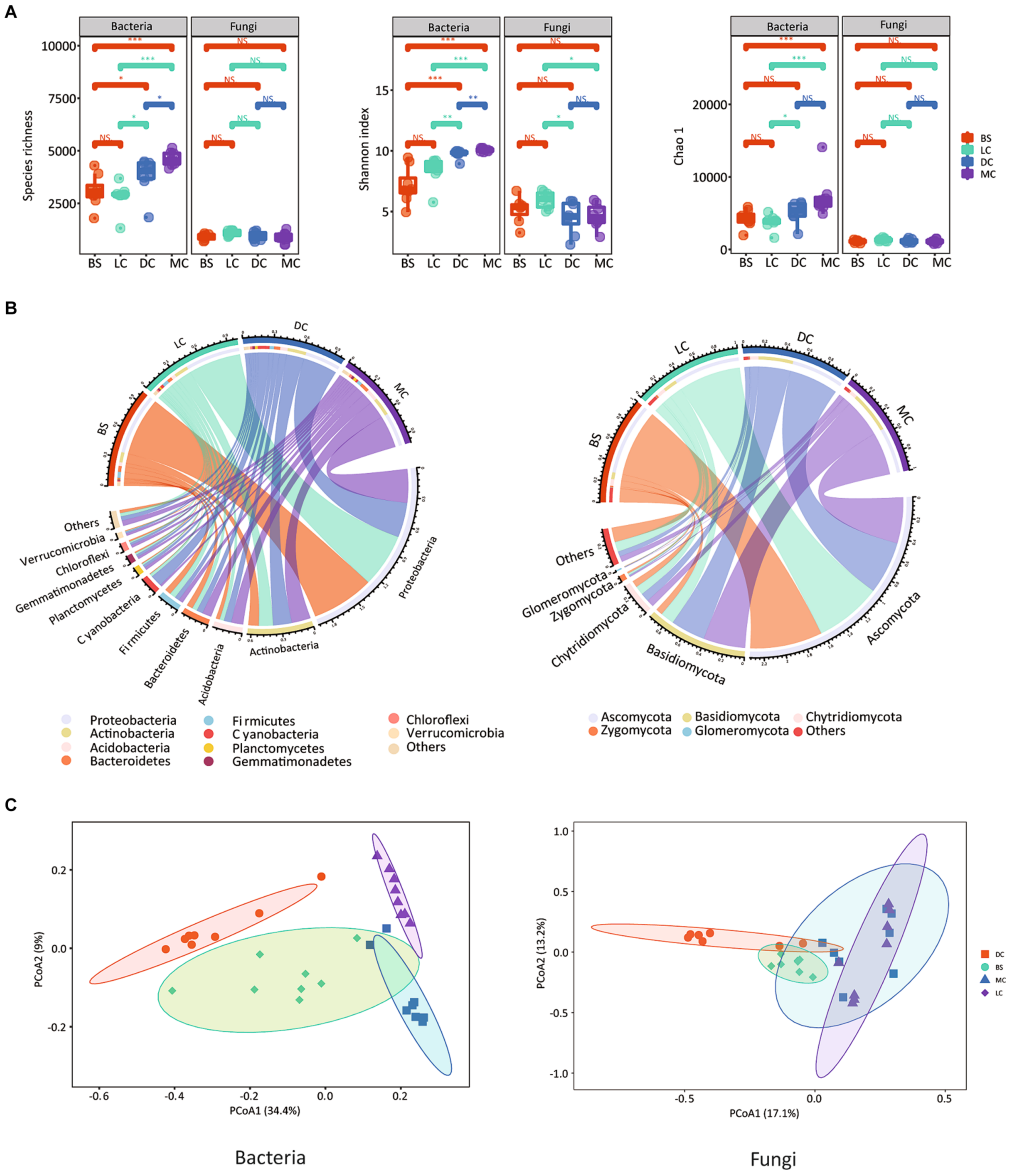
Alpha diversity of bacterial and fungal communities. **(A)** The lower 1/4 to upper 1/4 quantiles were visually depicted using box plots. Whiskers above and below the boxes represent the upper and lower 95% confidence intervals (CI), respectively. Outliers were visually represented by black solid circles above and below the boxes. Medians were represented using black horizontal bars within the boxes. Letters indicate significant differences between the biocrust types (*p* < 0.05); Relative abundances of the microbial phyla in the bacterial (left) and fungal (right) communities. **(B)** Bray-Curtis-based PCoA plots of the bacterial (left) and fungal (right) communities. **(C)** BS, LC, DC, and MC denote bare sand, light cyanobacterial biocrusts, dark cyanobacterial biocrusts, and moss crusts, respectively.

There were no significant differences in bacterial community diversity between bare sand and light cyanobacterial biocrusts (Figure 1A). However, notable variations in bacterial community diversity were observed among 3 biocrust types (Figure 1A). We observed gradual increases in the bacterial alpha diversity indices, namely Shannon diversity and species richness, over the biocrust development, with the highest values recorded in moss crusts. Specifically, Shannon diversity increased from 7.15 to 10.1 in bare sand and moss crusts, respectively (Figure 1A). Unlike the bacterial community, similar fungal diversities were observed in the bare sand and biocrust types. Bray-Curtis distance-based PCoA results indicated distinct bacterial and fungal communities among the four stages (Figure 1C), consistent with the PERMANOVA analysis findings, which revealed significant differences in bacterial and fungal communities among the four stages, with R^2^ values of 0.422 (*p* = 0.001) and 0.382 (*p* = 0.001) for bacteria and fungi, respectively (Supplementary Table S2).

In this study, we identified six dominant bacterial and four dominant fungal phyla in all samples. The bacterial phyla, based on their relative abundances, were predominantly Proteobacteria (47.8 ± 14.7%), Actinobacteria (15.8 ± 4.1%), Cyanobacteria (4.6 ± 5.0%), Acidobacteria (7.1 ± 2.1%), Bacteroidetes (7.1 ± 2.2%), and Firmicutes (5.6 ± 1.2%) (Figure 1B). Notably, the relative abundance of Proteobacteria in the bare sand exceeded that in the other stages, comprising 61.46, 54.99, 37.81, 35.89% in the bare sand, light cyanobacterial biocrusts, dark cyanobacterial biocrusts and moss crusts, respectively (Supplementary Table S3). In addition, the bare sand and light cyanobacterial biocrusts had higher relative abundances of Firmicutes, compared to the dark cyanobacterial biocrusts and moss crusts (*p* < 0.05). In the dark cyanobacterial biocrusts, the relative abundance of Cyanobacteria was significantly higher, accounting for 11.52%. The moss crusts, in contrast to other stages, exhibited the highest abundance of Actinobacteria. At the genus level, a total of 875 genera were identified among the four stages. Notably, the bare sand had a higher relative abundance of two proteobacterial genera, *Ralstonia* and *Delftia*, compared to the other stages. The dark cyanobacterial biocrusts showed significantly higher abundances of two cyanobacterial genera, *Microcoleus* and *Nostoc*, than the other stages (*p* < 0.05). Among the four developmental stages, the moss crusts had the highest abundance of five genera, namely *RB41*, *Bryobacter*, *Blastocatella*, *Nocardioides*, and *Pseudonocardia* (Supplementary Table S3). Ascomycota emerged as the dominant fungal group across the four stages (Figure 1B). Similar to bacterial communities, fungal communities showed significant differences among the four developmental stages. The relative abundance of Ascomycota in the bare sand and light cyanobacterial biocrusts was higher than that in dark cyanobacterial and moss crusts (Supplementary Table S3). The relative abundance of Basidiomycota was significantly higher in moss crusts. Additionally, the highest relative abundances of Ascomycota genus *Alternaria* was found in the bare sand, while the Ascomycota genus *Phoma* and Basidiomycota genus *Omphalina* were most abundant in moss crusts.

**Figure 2:**
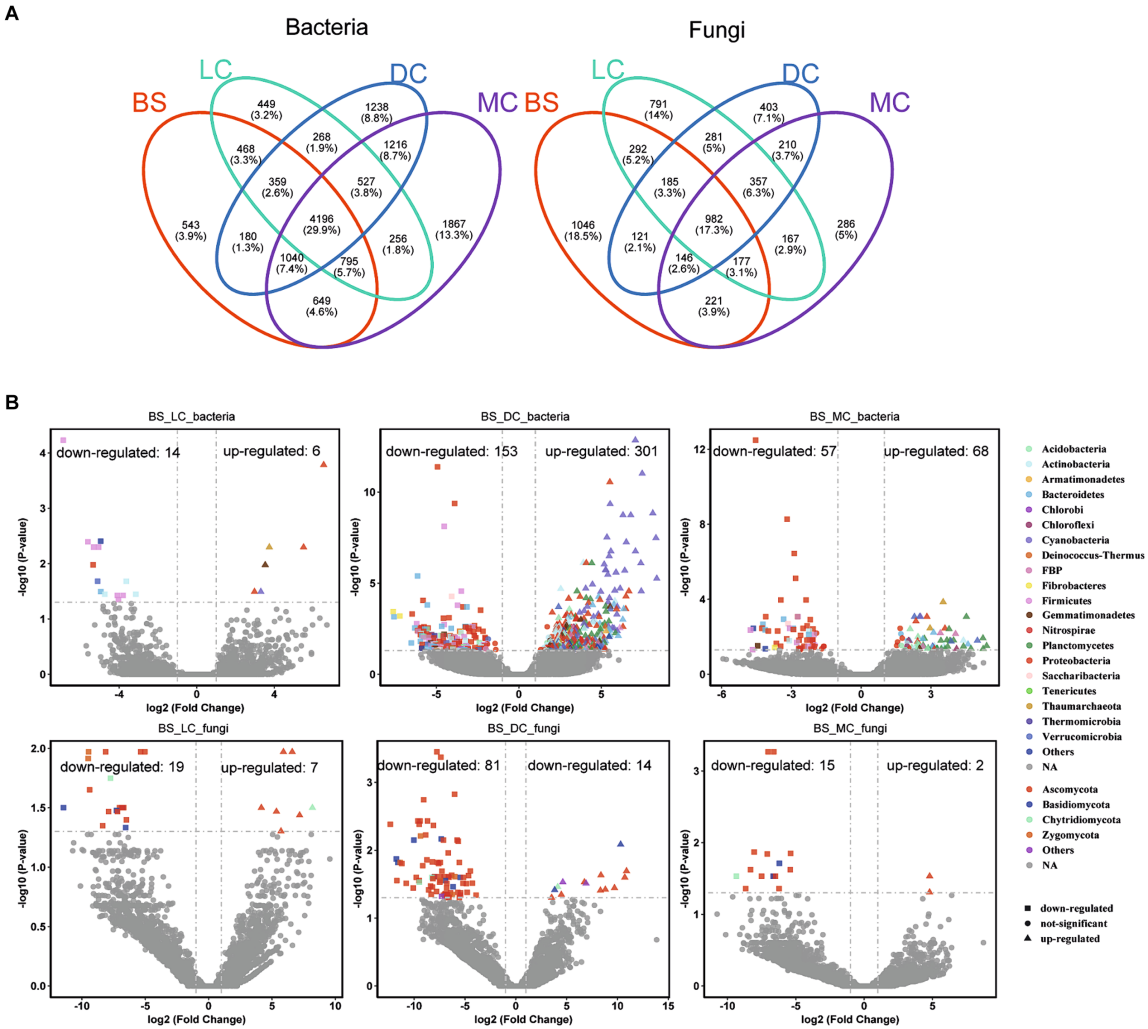
Number of OTUs of the bacterial and fungal communities in the different biocrust types. **(A)** Enriched and depleted bacterial and fungal OTUs in the light cyanobacterial biocrusts, dark cyanobacterial biocrusts, and moss crusts compared to the bare sand. **(B)** Each point represents an individual OTU, while the position along the y-axis represents the fold change in abundance. BS, LC, DC, and MC denote bare sand, light cyanobacterial biocrusts, dark cyanobacterial biocrusts, and moss crusts, respectively.

On the other hand, a total of 11,320 bacterial OTUs and 5,664 fungal OTUs were detected in bare sand and 3 biocrust types, with the majority exhibiting high abundance values (Figure 2A). Notable variations in OTU abundance were observed among the four different developmental stages. Additionally, the shared bacterial OTUs accounted for 37.1% of the total number between bare sand and biocrust types. The distinct bacterial OTUs in the bare sand, light cyanobacterial biocrusts, dark cyanobacterial biocrusts, and moss crusts accounted for 4.8, 4.0, 10.9, and 16.5% of the total number, respectively. In contrast to bacteria, the unique fungal OTUs gradually decreased over the development of biocrusts, showing 18.5, 14.0, 7.1, and 5.0% of the total number in the bare sand, light cyanobacterial biocrusts, dark cyanobacterial biocrusts, and moss crusts, respectively. However, the shared OTUs among four developmental stages accounted for only 17.3%. In this study, we performed differential abundance analyses using a negative binomial distribution with OTU counts. Bare sand was set as the control group, with an adjusted *p*-value cutoff of 0.05. According to the obtained results, the highest enriched and depleted bacterial OTU numbers were observed in the dark cyanobacterial biocrusts (301–153), followed, respectively, by those in the moss (68–57) and light cyanobacterial biocrusts (6–14) (Figure 2B). Indeed, the enriched bacterial OTUs in the dark cyanobacterial biocrusts were mainly derived from Cyanobacteria. Similarly, the highest enriched and depleted fungal OTUs were also observed in the dark cyanobacterial biocrusts (14–81), compared to those in the light cyanobacterial and moss crusts (Figure 2B). The depleted fungal OTUs in the dark cyanobacterial biocrusts were mainly derived from Ascomycota.

### 3.3 Ecological community assembly processes

**Figure 3:**
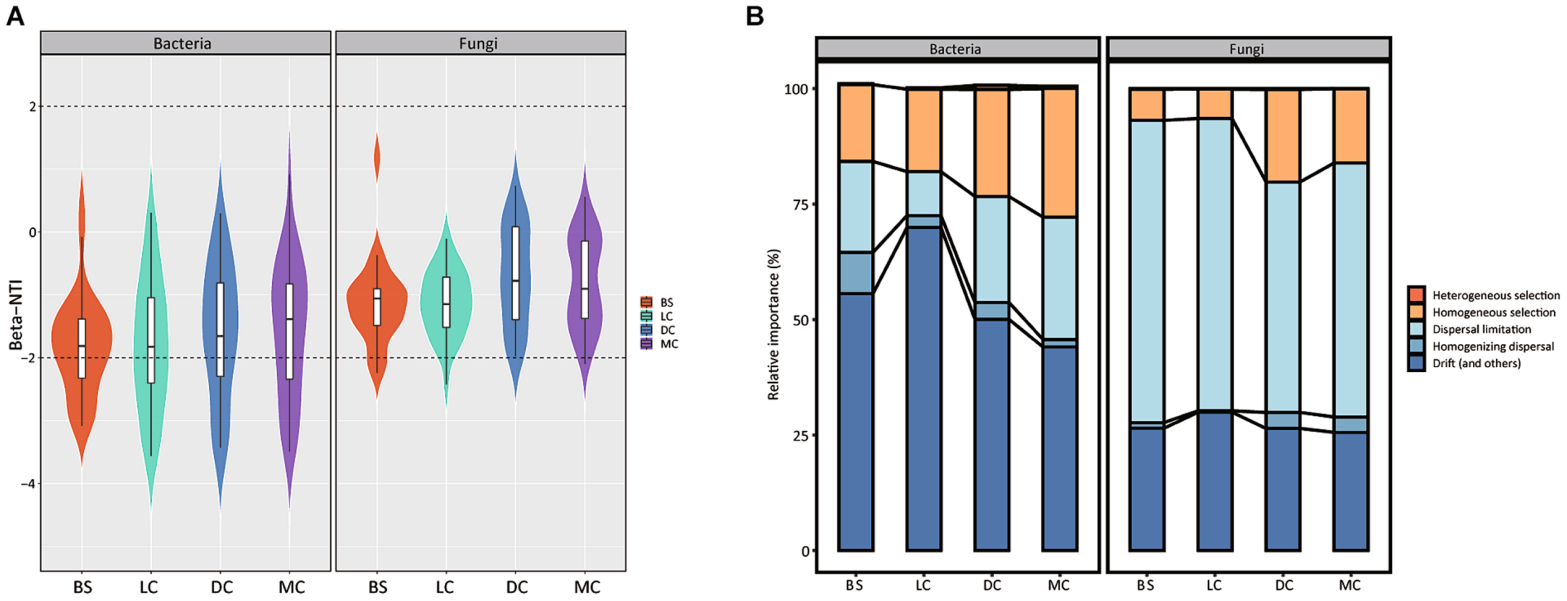
Deterministic and stochastic processes in microbiome assembly. **(A)** BS, LC, DC, and MC denote bare sand, light cyanobacterial biocrusts, dark cyanobacterial biocrusts, and moss crusts, respectively. Distributions patterns of betaNTI in the different biocrust types and the relative abundances of the bacterial and fungal communities. The percentage above and below the violin plot represent the proportion of the deterministic processes and stochastic processes in microbiome assembly, respectively. **(B)** The relative importance of five ecological processes. Each observation represents the standard deviation value and its associated null distribution. For both metrics, individual values below −2 and above +2 are statistically significant.

To determine the assembly patterns of bacterial and fungal communities across diverse biocrust types, we calculated the betaNTI index for paired biocrust samples. The majority of the betaNTI values for both bacterial and fungal communities in our study ranged from −2 to +2 (Figure 3A), suggesting the prevalence of neutral processes across all stages. Furthermore, we employed the iCAMP to quantify the relative contributions of ecological processes influencing the assembly of bacterial and fungal community assemblies. Our findings demonstrated that stochastic processes, specifically drift and dispersal limitation, were the primary drivers (>70% on average) shaping bacterial and fungal community assemblies across bare sand and 3 biocrust types. Notably, while bacterial community assembly was predominantly influenced by drift and dispersal limitation, fungal community assembly exhibited heterogeneous aggregation driven by similar processes. We observed a noticeable increase in deterministic processes (homogeneous selection) in the bacterial community with biocrust development, rising from 16 to 28%. Furthermore, the relative quantification of drift and dispersal processes governing bacterial and fungal community assemblies exhibited irregular changes with biocrust development, with some extreme and low values observed in light and dark cyanobacterial biocrusts, respectively.

### 3.4 Driving effects of physicochemical properties on biocrust microbial communities

**Figure 4:**
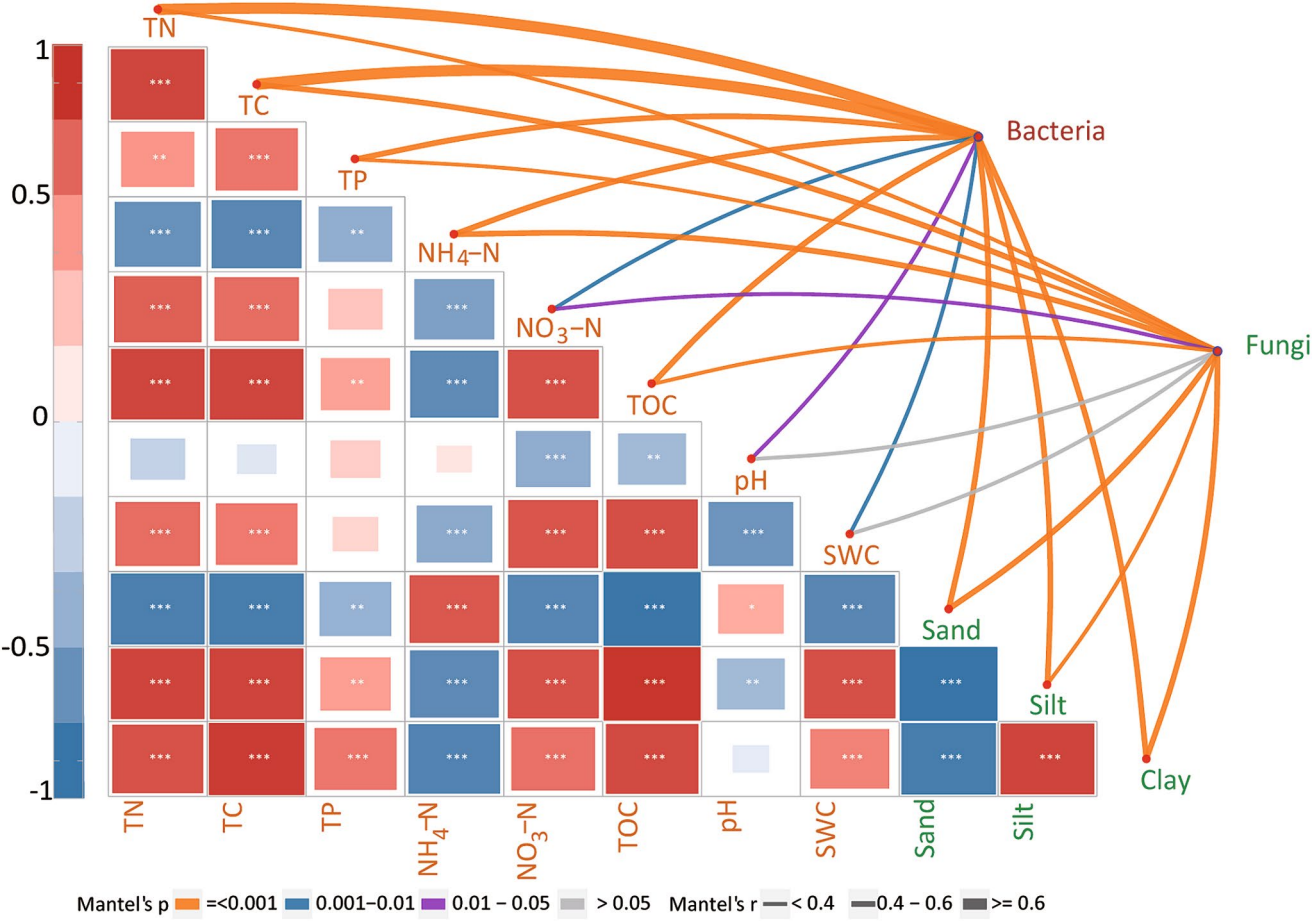
Mantel’s correlations between the environmental factors and biocrust microbial compositions. The widths of lines represent the magnitudes of Mantel’s r correlations, while the colors indicate the significance of the correlation results; the sizes of the colored blocks indicate the significance of the Pearson correlation between two environmental factors. SWC, TOC, TC, TN, TP, NO_3_^−^, and NH_4_^+^ denote soil water content, total organic carbon, total carbon, total nitrogen, total phosphorus, nitrate nitrogen, and ammonium nitrogen, respectively. *, **, and *** indicate significant results at the *p* < 0.05, *p <* 0.01, and *p <* 0.001 levels, respectively.

**Figure 5:**
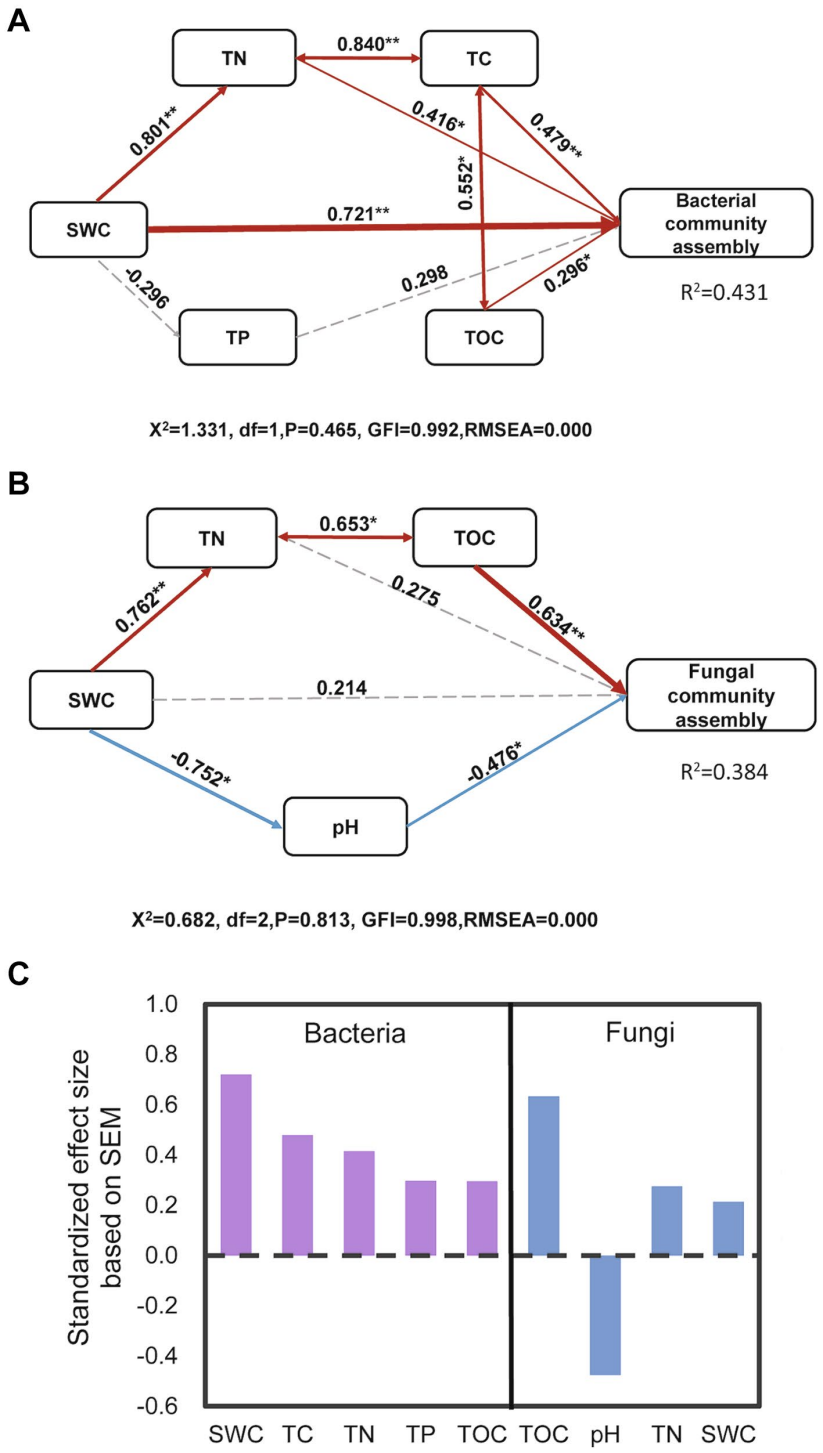
Structural equation modeling showing the relationships between environmental factors and the bacterial **(A)** and fungal **(B)** community compositions. Solid arrows indicate positive effects, and the dashed arrow indicates a negative correlation. The standardized path coefficients are adjacent to the arrows and indicate the effect size of the relationship. Arrow widths are proportional to the strength of each relationship. Percentages beside the response variables refer to the proportion of variance explained by the model (R^2^). Direction of total effects of environmental factors on bacterial and fungal community assembly in biocrusts. **(C)** SWC, TOC, TC, TN, and TP denote soil water content, total organic carbon, total carbon, total nitrogen, and total phosphorus, respectively. *, and ** indicate significant results at the *p* < 0.05, and *p <* 0.01 levels, respectively.

The Mantel test confirmed the significant effects of TN, TC, NH_4_^+^, SWC, TOC, sand, silt, clay, NO_3_^−^, TP, and pH values on the bacterial community compositions during biocrust development (Figure 4). Regarding the fungal community, the community composition showed significant correlations with TC, TOC, TP, TN, NO_3_^−^, sand, and clay contents (Figure 4). Since disentangling the ecological drivers controlling community assembly is crucial, we further developed the structural equation model (SEM) to explore the association of microbial community assembly with multiple factors (Figure 5). The model for bacterial community explained 43.1% of the variation in bacterial community assembly across biocrust development stages (Figure 5A). We found that SWC, TC, TN, and TOC emerged as significant factors shaping the bacterial community structure. Standardized total effects (including both direct and indirect effects) obtained from standardized SEM, suggested that SWC was the pivotal factor modulating variations in bacterial community assembly (Figure 5C). The model for fungi explained 38.4% of the variation in fungal community assembly with biocrust development (Figure 5B). Notably, the model indicated that soil TOC and pH values had significant direct effects on the assembly pattern of the fungal community (Figure 5C). Indirectly, SWC had positive effects on the variation of the fungal community, by enhancing TN and diminishing pH value during biocrust development (Figure 5B).

## 4 Discussion

### 4.1 Increased bacterial diversity and the abundance of copiotrophic bacteria along with the development of the biocrusts

The findings of the present study revealed a gradual increase in the α-diversity index of the biocrust bacterial community (Figure 1A), which is consistent with findings from previous studies (Mogul et al., 2017; Zhang B. C. et al., 2018). This observed phenomenon could be attributed to two principal factors: (1) the α-diversity of bacteria is primarily affected by the thickness of biocrusts during their development (Su et al., 2020). As biocrusts progress through various successional stages and increase in thickness, they provide a more stable habitat within the crust layer, thereby facilitating the enrichment of bacterial diversity. (2) the overall carbon and water content of the system exerts a dominant influence on bacterial diversity (Wang et al., 2018). As biocrusts develop, the enhanced availability of nutrients and water content (Supplementary Table S1) stimulate bacterial diversity, leading to a richer and more diverse bacterial community. In contrast, the diversity of fungal community remained relatively stable over biocrust development (Figure 1A). The disparities in the bacterial and fungal diversity could be attributed to fungi’s strong ability to withstand niche differentiation and water stress through their extensive hyphae (Jackson, 2012; Rousk et al., 2010).

In this study, the composition of the bacterial community changed significantly with biocrust development (Supplementary Table S3). For instance, oligotrophic groups that exhibit high stress tolerance, such as Proteobacteria and Firmicutes (Barnard et al., 2013), were more abundant in bare sand and light cyanobacterial biocrusts (Barnard et al., 2013). Notably, the Proteobacteria phylum can secrete extracellular polysaccharides to bind sand grains, exhibiting strong N fixation capacities under limited N conditions (Gundlapally and Garcia-Pichel, 2006; Maier et al., 2018; Pepe-Ranney et al., 2016). Consequently, a high abundance of Proteobacteria may play a crucial role in stabilizing the soil surface and providing adequate nutrients for biocrust formation. In contrast, significant increases in the relative abundances of the copiotrophic groups (e.g., Actinobacteria) were observed during biocrust development (Supplementary Table S3). This proliferation is likely attributed to improved soil nutritional conditions. It is worth noting that most Actinobacteria are mycelial, which potentially contributes significantly to maintaining the structure of mature biocrust types (Maier et al., 2016). Cyanobacteria, important photosynthetic autotrophs within biocrusts, are capable of fixing inorganic carbon and nitrogen from the atmosphere (Rong et al., 2022). Our findings revealed an increase in the relative abundance of Cyanobacteria in dark cyanobacterial biocrusts, whereas a decrease was observed in moss crusts. This trend aligns with previous research (Zhang et al., 2016) and may be explained by the dense pseudo-roots and thickness of bryophyte mats in moss crusts, which potentially alter the habitat for Cyanobacteria.

Ascomycetes were the dominant fungal groups in the bare sand and light cyanobacterial biocrusts in the desert regions, followed by Basidiomycetes and Chytridiomycota, respectively (Figure 1B; Supplementary Table S3). This pattern is consistent with previous findings from the Sultanate of Oman and the Gurbantunggut Desert (Abed et al., 2013; Zhang B. C. et al., 2018). The prevalence of Ascomycota can be attributed to their melanin-rich cell walls, which protect biocrusts against harsh environmental conditions like desiccation and intense UV radiation, particularly in early biocrust stages (Gostincar et al., 2010). The relative abundance of Basidiomycetes in dark cyanobacterial and moss crusts exceeded significantly that in bare sand and light cyanobacterial biocrusts (*p* < 0.05) (Supplementary Table S3). Basidiomycetes play a role in degrading recalcitrant materials, such as lignin (Zhu et al., 2016). Therefore, the changes in the fungal phyla suggested alterations in the fungal community functions throughout the biocrust development. The fungal community in early biocrust types may primarily withstand environmental stressors, subsequently transitioning to facilitate organic matter degradation and nutrient accumulation.

### 4.2 The contribution of the deterministic processes increases along with the development of the biocrusts

In our study, both deterministic and stochastic processes jointly influenced the assembly of bacterial and fungal communities within biocrusts, with stochastic processes (specifically, dispersal limitation and drift) predominating (>70% on average, Figure 3B). This finding is consistent with previous studies that have highlighted the significance of stochastic assembly under extreme environments (Gao et al., 2020; Marasco et al., 2018; Verbruggen et al., 2012). The dominance of stochastic processes in biocrust could be attributed to two main reasons. Firstly, previous study has shown that an increase in the aggregation of K-strategists could enhance stochastic process (Guo et al., 2022). In our study, the relative abundances of Actinobacteria and Acidobacteria phyla, which primarily consist of K-strategists (Gu et al., 2018; Lee et al., 2008), increased with the biocrust development (Figure 1B). Secondly, the enhancement of soil nutrients (e.g., TC, TOC, TN, and TP) with biocrust development could lead to strong selection pressure, resulting in an increased relative importance of deterministic processes but a decreased importance of stochastic processes (Figure 3A). This explanation is supported by a recent study, which emphasized the importance of stochastic assembly under low nutrient conditions, as no species can persist or gain any advantage in harsh habitats (Ning et al., 2024). Furthermore, it is worth noting that the proportion of deterministic processes increased in shaping bacterial community structure (Figure 3A), whereas it remained stable in the fungal community structure (Figure 3B) with the biocrust development. One possible reason is that bacteria were more susceptible to deterministic processes (e.g., environmental filtration) than fungi, as previously demonstrated (Dini-Andreote et al., 2015; Huo et al., 2023; Tripathi et al., 2018). This is due to the fact that bacteria tend to have faster generation times and higher mobility, which enable them to utilize various resources in a competitive environment and effectively adapt to harsh conditions (Schmidt et al., 2014; Sun et al., 2017; Wang et al., 2013). On the contrary, fungal members typically have larger size and weaker dispersal abilities compared to bacteria (Chen et al., 2017). Thus, fungi are more susceptible to dispersal limitation (Wang et al., 2021).

### 4.3 The key environmental factors for the assembly of bacteria and fungi, SWC for bacteria, while TOC for fungi

The Mantel test revealed significant correlations between bacterial community composition and TN (*p <* 0.001), TC (*p* = 0.001), SWC (*p <* 0.001), sand content (*p <* 0.001), silt content (*p* = 0.009), TOC (*p* = 0.001), TP (*p* = 0.001), NO_3_-N (*p* = 0.001), NH_4_-N (*p* = 0.001), and pH value (*p* = 0.004) throughout the biocrust development (Figure 4). These results suggested that variations in bacterial community composition in biocrusts were influenced by the soil nutrients and water along the biocrust development. Similar to above results, SEM analysis showed that SWC, TC, TN and TOC exerted significantly positive effects on bacterial community assembly (Figure 5A). Particularly, SWC emerged as the most influential factor (*p* = 0.001, λ = 0.721; Figure 5C). This finding aligns with the results in Qinghai-Tibet Plateau desert, similarly identified SWC as the primary factor influencing bacterial community assembly in biocrusts (Ding et al., 2023). This phenomenon could be attributed to several possible reasons: Firstly, higher soil water content may stimulate bacterial activity and increase their biomass in desert soils (Nielsen and Ball, 2015; Sorensen et al., 2013); Secondly, elevated soil water content might enhance the mineralization of soil organic matter (Austin et al., 2004; Grigg et al., 2008; Zhang et al., 2022), thereby providing more nutrients to the bacterial community. Lastly, increases in soil water content can accelerate bacterial growth, resulting in greater differentiation within bacterial community, as demonstrated in previous study (Cruz-Paredes et al., 2021). These factors, either individually or in combination, contribute to the assembly of the bacterial community within biocrusts.

Furthermore, the results of the mantel test showed that fungal community composition of biocrusts was significantly influenced by TOC (*p <* 0.001), TC (*p <* 0.001), TN (*p* = 0.001), sand content (*p* = 0.001), clay content (*p* = 0.001) and NO_3_-N (*p* = 0.003), but showed no significant correlations with SWC (*p* = 0.007; Figure 4). Similarly, SEM analysis emphasized TOC as the most critical factor affecting fungal community assembly (*p <* 0.001, λ = 0.634; Figures 5A,C). It is unsurprising, as previous studies have consistently identified TOC as the primary factor influencing fungal community composition and interactions within biocrusts (Zhang Q. Y. et al., 2018; Zhou et al., 2020). Given that the majority of fungi are saprotrophic and directly involved in decomposing soil organic matter, TOC content is closely linked to fungal community assembly (Shah et al., 2016). Additionally, SEM results also indicated that soil pH had significant direct negative effects on the fungal community assembly (*p =* 0.037, λ = −0.476; Figures 5A,C). This finding aligns with previous studies demonstrating soil pH was the main environmental driver affecting soil fungal community assembly in temperate desert of northern China (Chen et al., 2017; Yang et al., 2017). Previous study has shown that the optimum pH for most of fungus growth is narrow, typically ranging between 5 and 7 units (Rousk et al., 2009). When soil pH exceeds their optimum growth pH, fungal communities become more sensitive to pH changes (Wheeler et al., 1991). In our study, all soil samples had a pH greater than 7 (Supplementary Table S1), highlighting the essential role of soil pH in soil fungal community assembly within biocrusts. Moreover, unlike bacteria, SWC was not the primary factor influencing fungal community assembly. This divergence could be attributed to fungi’s strong ability to access soil water through their extensive hyphae (Jackson, 2012).

## 5 Conclusion

In this study, we comprehensively assessed bacterial and fungal diversity patterns, as well as the assembly mechanisms and drivers shaping their microbial communities, across different biocrust successional stages in the Mu Us Sandy Land. Our findings highlight how biocrust development strongly alters microbial community patterns. While stochastic processes mainly governed the assembly of bacterial and fungal communities within biocrusts, deterministic factors increasingly influenced the formation of bacterial communities along the biocrust successional stages. The variation in microbial communities across different biocrust successional stages was driven by a combination of biocrust physicochemical properties, with soil water content (SWC) emerging as the most crucial factor affecting bacterial community assembly, while total organic carbon (TOC) played a pivotal role in fungal community assembly. Additionally, by distinguishing stochastic and deterministic processes and their relative importance in different biocrust developmental stages, we provide a deep understanding of microbial community assembly in extreme environments.

## Data Availability

The datasets presented in this study can be found in online repositories. The names of the repository/repositories and accession number(s) can be found in the article/Supplementary material.

## Footnotes

^1^https://cran.r-project.org/
