# Supplementary material for: Deterministic processes influence bacterial more than fungal community assembly during the development of biological soil crusts in the desert ecosystem
Source: Front Microbiol. 2024 Aug 23;15:1404602. doi: 10.3389/fmicb.2024.1404602 (PMC11377341; doi:10.3389/fmicb.2024.1404602)
Supplement: Supplementary file 1 [file Data_Sheet_1.pdf]

Supplementary Material

1. Deterministic processes influence bacterial more than fungal community assembly during the development of biological soil crust in the desert ecosystem

Hong Zhou[1,2,3,5,†], Ke Yu[6,†], Chunfang Deng[6], Bo Wu[2,4,5], Ying Gao[2,4,5*]

*** Correspondence:** Ying Gao: yinggao@caf.ac.cn;

1 Supplementary Figures and Tables

1.1 Supplementary Tables

Supplementary Table S1. Chemical and physical features of biocrusts

	Bare sand	Light cyanobacterial biocrusts	Dark cyanobacterial biocrusts	Moss crusts
SWC (%)	0.23±0.10d	0. 41±0.10c	0.43±0.10c	1.15±0.10a
pH	7.23±0.05a	7.19±0.04a	7.12±0.03a	7.03±0.01b
TOC ($g\cdot kg^{-1}$)	0.62±0.15d	6.03±0.84c	9.12±0.65b	14.99±0.66a
TC ($g\cdot kg^{-1}$)	2.16±0.13c	13.50±1.81b	17.16±2.15a	19.82±1.70a
TN ($g\cdot kg^{-1}$)	0.28±0.12d	0.68±0.11c	0.97±0.13b	1.12±0.10a
NO_3-N (10^{-4} $g\cdot kg^{-1}$)	6.51±1.12a	3.39±0.96b	3.12±1.03b	2.05±0.63c
NH_4-N (10^{-4} $g\cdot kg^{-1}$)	3.92±2.29c	4.81±0.65c	5.59±0.81b	7.04±0.46a
TP ($g\cdot kg^{-1}$)	0.38±0.10b	0.64±0.04a	0.62±0.05a	0.58±0.06a

Sand (%)	95.36±0.19a	88.96±0.14b	86.64±0.07b	81.00±0.04c
Silt (%)	4.12±0.07c	9.46±0.02	11.72±0.07	17.10±0.12
Clay (%)	0.52±0.06c	1.55±0.02b	1.64±0.04a	1.90±0.08a
MBC ($mg \cdot kg^{-1}$)	66.07±16.45d	139.21±31.78c	278.42±35.92b	389.80±26.16a
MBN ($mg \cdot kg^{-1}$)	5.71±1.22d	13.59±2.24c	30.32±2.21b	39.42±4.36a

*Values represent means ± standard errors (n = 3). Different letters indicate significant differences among developmental stages at $P<0.05$.

*Abbreviations: SWC, soil water content; TOC, total organic carbon; TC, total carbon; TN, total nitrogen; TP, total phosphorus; NO_3-N, nitrate nitrogen; NH_4-N, ammonium nitrogen.

Supplementary Table S2. PerMANOVA (pairwise comparisons between treatments) to evaluate variations in the bacterial and fungal community structure. Significant P values are in bold.

Groups	Bacteria		Fungi	
	R^2	*P*	R^2	*P*
BS-LC	0.262	**0.005**	0.486	**0.002**
BS-DC	0.309	**0.001**	0.528	**0.005**
BS-MC	0.461	**0.001**	0.543	**0.002**
LC-DC	0.274	**0.001**	0.399	**0.013**
LC-MC	0.323	**0.001**	0.452	**0.001**
DC-MC	0.221	**0.002**	0.128	**0.025**
BS-LC-DC- MC	0.422	**0.001**	0.382	**0.034**

BS = bare sand; LC = Light cyanobacterial biocrusts; DC = Dark cyanobacterial biocrusts; MC = moss crusts.

Supplementary Table S3. Results of one-way ANOVA of the relative abundances of microbial phyla and genera (≥1%) of different developmental stages of BIOCRUSTs. The mean value and standard error (n = 3) of are shown in the right columns of the table. P values reflecting statistical significance are shown in boldface. Lowercase letters in the right columns of the table indicate significant differences observed following water addition ($P< 0.05$).

**Domain**	**Taxa groups**	**Bare sand**	**Light cyanobacterial biocrusts**	**Dark cyanobacterial biocrusts**	**Moss crusts**
Bacteria	*p_Actinobacteria*	9.46±1.63**c**	12.64±2.49**b**	19.07±2.27**a**	19.89±1.31**a**
	p_Acidobacteria	3.27±0.83**d**	5.35±0.85**c**	7.65±0.61**b**	11.87±1.61**a**
	p_Bacteroidetes	6.37±0.71**c**	5.76±1.22**c**	5.64±0.06**b**	10.44±1.16**a**
	p_Gemmatimonadetes	1.07±0.17**c**	2.25±0.19**b**	2.66±0.21**b**	3.25±0.15**a**
	p_Planctomycetes	0.84±0.14**c**	1.88±0.18**b**	2.86±0.24**a**	4.17±0.53**a**
	p_Cyanobacteria	0.70±0.17**d**	3.38±0.65**c**	11.52±0.96**a**	1.77±0.70**c**
	p_Proteobacteria	61.46±7.51**a**	54.99±3.52**b**	37.81±1.45**c**	35.89±2.79**c**
	p_Firmicutes	7.41±0.87**a**	6.13±0.17**a**	5.06±0.38**b**	3.35±0.59**b**
	p_Chloroflexi	2.41±0.22	2.12±0.13	2.33±0.24	2.37±0.25
	g_Ralstonia	31.6±8.6**a**	15.2±6.5b	1.6±0.7c	2.3±0.6c
	g_Delftia	5.6±0.7**a**	3.4±0.4b	0.6±0.3c	1.0±0.2c
	g_RB41	0.8±0.4**c**	0.9±0.5c	1.5±0.4c	3.8±1.3a
	g_Microcoleus	0.1±0.1d	1.0±0.4c	3.8±1.0a	0.3±0.1d
	g_Bryobacter	0.5±0.1c	0.5±0.2c	0.6±0.1b	1.3±0.2a
	g_Nocardioides	0.6±0.1c	0.6±0.2bc	0.8±0.2b	1.3±0.2a
	g_Pseudonocardia	0.2±0.1b	0.3±0.1b	1.1±0.2a	0.8±0.2a
	g_Blastocatella	0.2±0.1c	0.3±0.1c	0.7±0.1b	1.0±0.1a
	g_Nostoc	0.1±0.1c	0.1±0.1c	7.9±0.3a	0.1±0.1c
	p_Basidiomycota	7.19±0.97**b**	12.31±2.24**b**	34.04±3.61**a**	41.00±0.73**a**
Fungi	*p__Ascomycota*	76.40±2.10**a**	66.38±2.56**a**	56.46±6.39**b**	48.04±2.58**b**
	p_Chytridiomycota	5.51±0.96**a**	13.58±4.73**a**	6.36±0.41**a**	6.95±0.59**a**

	p_Zygomycota	4.54±0.24**a**	2.82±0.35**a**	1.53±0.14**a**	0.98±0.31**a**
	g_Alternaria	24.1±3.9a	4.2±1.5b	0.8±0.3c	1.1±0.6c
	g_Phoma	0.4±0.2b	0.4±0.5b	12.3±4.6a	17.5±4.2a
	g_Omphalina	0.1±0.1c	0.1±0.1c	0.4±0.2b	4.7±1.1a
	g_Laetisaria	0.1±0.1b	0.1±0.3b	9.9±2.6a	0.4±0.3b
	p_Thaumarchaeota	70.22±5.42	73.2±4.51	72.43±3.67	73.19±7.32
	p_Euryarchaeota	4.36±1.24	4.52±2.03	3.87±2.42	3.92±2.06
Archaea	*p_unidentified*	1.14±1.52	2.25±1.59	1.56±0.71	1.95±0.83
	g_Soil_Crenarchaeotic_Group	18.33±5.35	20.24±6.39	18.88±7.03	17.20±6.15
	g_Candidatus_Nitrososphaera	0.43±0.22	0.62±0.47	0.93±0.61	0.75±0.42

1.2 Supplementary Figures

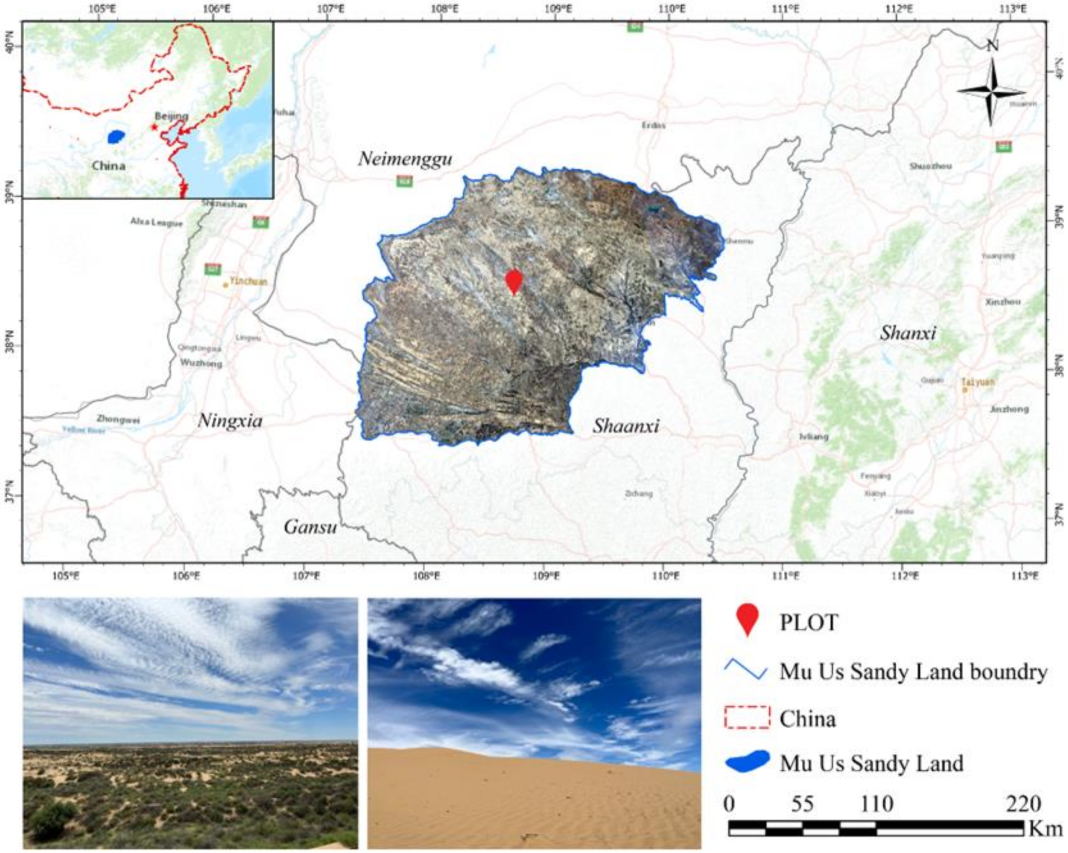

Supplementary Figure S1. Location of the study area. The lower left figure shows fixed and shifting sand dunes, respectively.

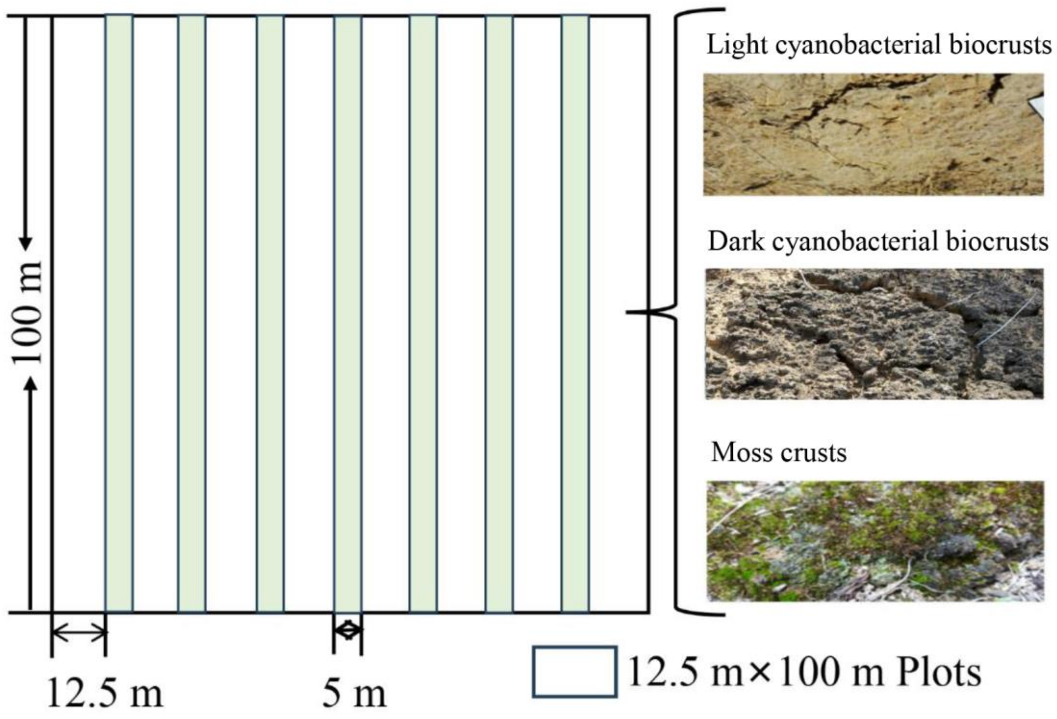

Supplementary Figure S2. Sample collection diagram. There were 3 types of biocrusts were randomly collected in 8 replicate plots (left). The white boxes indicate the different plots, and the green boxes indicate the spacing between plots.

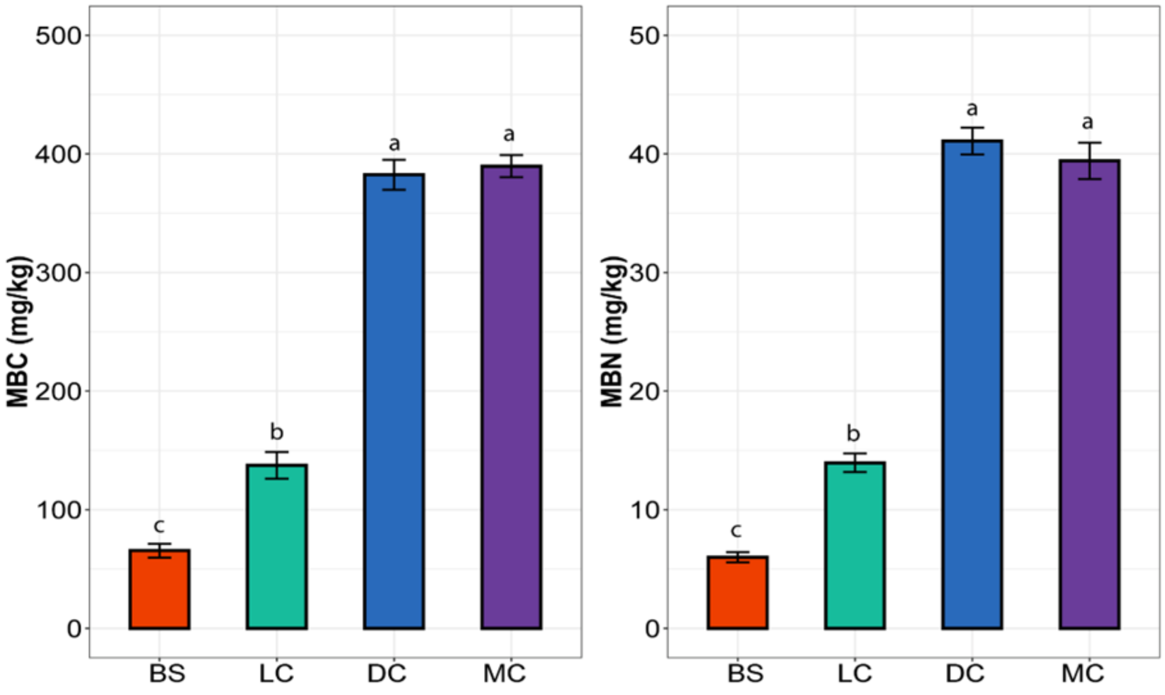

Supplementary Figure S3. Microbial biomass carbon (MBC) and microbial biomass nitrogen (MBN) of different developmental stages of biocrusts. Letters depict significant differences across different developmental stages of biocrusts. BS = bare sand; LC = Light cyanobacterial biocrusts; DC = Dark cyanobacterial crusts; MC = moss crusts.

1.3 Supplementary of materials and methods

Supplement of materials and methods. QIIME 2 was used to generate raw sequence data from sequencing, and all raw reads were aligned to samples according to different barcodes. Both forward and reverse primers were trimmed. Paired end reads of suffcient length were combined with at least 30 bp overlap into full length sequences, and the average fragment length was 253 bp using the FLASH program (Reyon et al., 2012). Unqualifed sequences were fltered using the Btrim program, with a window size >20 as the threshold Quality Score (Yong, 2011). UPARSE was used to remove chimeric sequences and classify sequences into operational taxonomic units (OTUs) at a similarity of 97% (Edgar et al., 2011). All singleton OTUs were removed. Bacterial representative sequences were assigned taxonomic information using the SILVA database as a reference (Pruesse, 2007) and fungal representative sequences were assigned taxonomic information using the UNITE database (Abarenkov et al., 2010). Finally, subsampling was performed to normalize the dataset to the sample with the lowest number of reads. Our amplicon sequencing detected a small number of archaeal sequence reads, rarefied to 12 965 sequences. Although these archaeal sequences were distributed in 88 OTUs and 3 phyla, they were unidentified at low classification levels. In addition, no significant differences in the archaeal phylum were observed between the biocrust types (Table. S3). Therefore, only bacterial and fungal communities were further studied in this study.

References

Abarenkov, K., Henrik, N.R., Larsson, K.H., Alexander, I.J., Eberhardt, U., Erland, S., Høiland, K., Kjøller, R., Larsson, E., Pennanen, T., Sen, R., Taylor, A., Tedersoo, L., Ursing, B.M., V, T., Liimatainen, K., Peintner, U., K, U. (2010). The UNITE database for molecular identification of fungi-recent updates and future perspectives. New Phytologist 186, 281-285.

Edgar, R.C., Haas, B.J., Clemente, J.C., Christopher, Q., Rob, K. (2011). UCHIME improves sensitivity and speed of chimera detection. Bioinformatics 27, 2194.

Pruesse, E., Quast, C., Knittel, K., Fuchs, B.M., Ludwig, W., Peplies, J., Glockner, F.O. (2007). SILVA: a comprehensive online resource for quality checked and aligned ribosomal RNA sequence data compatible with ARB. Nucleic Acids Research 35, 7188-7196.

Reyon, D., Tsai, S.Q., Khayter, C., Foden, J.A., Sander, J.D., Joung, J.K. (2012). FLASH assembly of TALENs for high-throughput genome editing. Nature Biotechnology 30, 460-465.

Yong, K. (2011). Btrim: a fast, lightweight adapter and quality trimming program for next-generation sequencing technologies. Genomics 98, 152-153.
